# Machine learning enhanced grey box soft sensor for melt viscosity prediction in polymer extrusion processes

**DOI:** 10.1038/s41598-025-85619-6

**Published:** 2025-02-15

**Authors:** Yasith S. Perera, Jie Li, Chamil Abeykoon

**Affiliations:** 1 Northwest Composites Centre and Aerospace Research Institute, Department of Materials, Faculty of Science and Engineering, The University of Manchester, Oxford Road, Manchester, M13 9PL UK; 2 Centre for Process Integration, Department of Chemical Engineering, School of Engineering, The University of Manchester, Manchester, M13 9PL UK

**Keywords:** Materials science, Engineering, Mathematics and computing

## Abstract

Melt viscosity is regarded as a key quality indicator of the polymer melt in polymer extrusion processes. However, limitations such as disturbances to the melt flow and measurement delays of the existing in-line and side-stream rheometers prevent the monitoring and controlling of this key parameter in real time. Soft sensors can be employed to monitor physical parameters that are difficult to measure using hardware sensing instruments. This study presents a grey-box soft sensing solution to predict the melt viscosity in real time, which combines physics-based knowledge with machine learning. A fine-tuned physics-based mathematical model is used to make melt viscosity predictions, and a deep neural network is employed to compensate for its prediction errors. The proposed soft sensor model reported a normalised root mean square error of 2.2$\:\times\:$10^−3^ (0.22%), outperforming fully data-driven soft sensor models based on multilayer perceptron and long short-term memory neural networks. Furthermore, it exhibited an improvement of approximately 95% in terms of predictive performance, compared to a soft sensor based on a radial basis function neural network reported in a previous study. The proposed soft sensor can monitor viscosity changes caused by changes in operating conditions but not suitable for detecting viscosity changes due to changes in material properties. The findings of this study can aid in enhancing process monitoring and control in polymer extrusion processes.

## Introduction

Polymer extrusion is a fundamental processing stage in producing a wide range of plastic products^1^. Melt viscosity is a key indicator of melt quality in continuous polymer extrusion processes. Consistency and homogeneity of the melt viscosity directly influence the functional, aesthetic, and dimensional properties of the extruded products^2^. Offline measurements result in a considerable time lag between the manufacturing of the product and the identification of quality issues, which ultimately leads to material waste^3^. Hence, precise control of melt viscosity during extrusion would enable the desired product quality to be achieved and maintained while minimising material waste. However, to realise this, real-time monitoring of the melt viscosity is necessary. The existing commercial polymer extruders are not equipped with any melt viscosity measuring instruments, which inhibits the implementation of real-time quality control measures.

Past researchers have investigated techniques such as in-line and side-stream (i.e., online) rheometers to measure the melt viscosity in real time, but these instruments also suffered from various limitations^3–9^. Side-stream rheometers can measure the melt viscosity during extrusion without disrupting the melt flow but suffer from significant time delays in the order of minutes^6^ and hence fail to capture the process dynamics accurately. In contrast, in-line rheometers can make real-time measurements without a delay but disturb the melt flow, while resulting in reduced throughput rates. These limitations render the in-line and side-stream rheometers incompatible with industrial polymer extrusion processes.

Ultrasound velocity profile with pressure differential has been a widely studied technique for in-line rheological measurements, which is non-invasive, inexpensive, and easy to install^10^. This technique employs ultrasound transducers that emit a series of short ultrasound pulses to obtain the velocity profile of a fluid by detecting the waves reflected by the moving fluid particles. This information is then used to estimate the melt viscosity. However, this technique is also associated with limitations such as inaccurate transducer measurements due to the effect of ultrasonic near-field, difficulty in estimating the ultrasound velocity along the beam axis, and the sensitivity of the determined rheological parameters to ultrasonic parameters^10^. Tasaka et al.^11^ proposed a non-intrusive in-line rheometric method based on ultrasonic spinning rheometry, which eliminates the need to measure the pressure difference. However, the viscosity range that can be measured is limited, and this technique has not been tested on industrial processes.

The limitations of these physical melt viscosity monitoring devices have rendered them unsuitable for real-time monitoring of melt viscosity in polymer extrusion processes. As a result, the melt quality is assessed offline, away from the extruder, using laboratory rheometers. This prevents the implementation of real-time melt viscosity control techniques^12^. Several previous studies have attempted to control the melt viscosity based on feedback obtained using in-line rheometer dies^13–17^. However, the use of an in-line rheometer makes them impractical for industrial polymer extrusion processes due to the flow constrictions and reduced production rates caused by the in-line rheometer die. Consequently, real-time melt viscosity monitoring has become necessary for improving process control in industrial polymer extrusion processes.

Soft sensors or virtual sensors are an attractive alternative for estimating physical parameters that are difficult to measure in real time using hardware sensors. Soft sensors have been used in applications across a wide range of industrial processes^18–23^. Soft measurement techniques have been investigated for estimating key parameters such as the melt temperature profile, melt viscosity, melt pressure, energy consumption, flow rate, and mechanical properties of the extrudate in industrial polymer extrusion processes as well^24–37^. The study by Kumar et al.^32^ is one of the earliest works that proposed a soft sensing approach for melt viscosity prediction. The soft sensor was based on a physics-based first-principles model. However, the model was derived based on several assumptions that could adversely affect its predictive performance. Moreover, the accuracy of the model depended on the accuracy of the feed rate and die pressure measurements. The work by Chen et al.^33^ is another early study that proposed an empirical model to predict the melt viscosity. However, the accuracy of the model was influenced by the consistency of the polymer melt properties.

McAfee and Thompson^34^ reported a soft sensor based on a grey-box modelling technique to predict the melt viscosity in a single-screw extruder. A linear-in-the-parameter polynomial model with a nonlinear autoregressive with exogenous input (NARX) model structure was used to construct two grey-box models in series. The first model (i.e., viscosity model) predicts the melt viscosity based on input process parameters (i.e., screw speed and barrel set temperatures), which in turn is fed to the second model (i.e., pressure model), that predicts the melt pressure at the die. The predicted die melt pressure is then compared with the actual die melt pressure measured using a hardware sensor, and the error between the predicted and measured values is used as feedback to correct the errors of the viscosity model. The grey-box model structure enabled providing insight into how the process parameters affected the melt viscosity. In another study, McAfee and Thompson^35^ introduced an online correction mechanism to make the soft sensor adaptive to changes in operating conditions and feed material. Later, Liu et al.^36^ proposed an improved version of the soft sensor reported in the previous work by McAfee and Thompson^34^. They used a nonlinear finite impulse response (NFIR) model structure instead of the complex NARX model structure reported in the previous study^34^. The model could be made adaptive to different polymeric materials and die designs by updating the model parameters online. In another study, Deng et al.^37^ proposed a data-driven soft sensor based on a radial basis function (RBF) neural network optimised using a differential evolution (DE) algorithm and a two-stage selection algorithm, to predict the melt viscosity in a single-screw extrusion process.

Although several past studies have attempted to develop soft sensors to predict the melt viscosity in real time, several limitations in these soft sensors can be identified. First-principles models were derived based on several assumptions and were not capable of capturing actual process dynamics. Early empirical models also suffered from poor predictive performance due to the use of conventional modelling algorithms. Despite the use of machine learning techniques, the soft sensor by Deng et al.^37^ reported a high root mean square percentage error (RMSPE) of 9.35%, and the residual plot results indicated errors with a magnitude as high as 500 on an unseen dataset. Soft sensors proposed by McAfee and Thompson^34^ and Liu et al.^36^ provide good prediction accuracy over a wide range of processing conditions, however, these works were based on traditional modelling techniques. In the existing literature, there is a gap in assessing the potential of modern deep learning methods and hybrid artificial intelligence-driven approaches to enhance the prediction accuracy of melt viscosity soft sensors. Therefore, there is room for further improvement in terms of predictive performance of these soft sensing solutions by integrating deep learning methods.

This study presents a soft sensor based on a grey-box modelling technique to predict the melt viscosity in a single-screw extruder in real time. A grey-box model architecture was chosen for the soft sensor, as grey-box models are generally expected to perform better than white-box and black-box models. A combined grey-box (CGB) model architecture^38^ that combines physics-based knowledge about the extrusion process with artificial intelligence-based techniques is proposed. The proposed CGB model is composed of a serial grey-box (SGB) component and a parallel black-box component. The SGB component comprises a physics-based model, the parameters of which were fine-tuned using linear regression. As the black-box component, a deep neural network was chosen. The SGB component predicts the melt viscosity while the black-box component estimates the prediction error of the SGB component. The prediction of the black-box component is then added to the SGB component to arrive at the final melt viscosity prediction. Although previous works have reported serial grey-box architectures^34–36^, no existing studies have proposed combined grey-box architectures to predict the melt viscosity in polymer extrusion processes.

Multilayer perceptron (MLP) neural networks have been a favourable candidate for many soft sensing applications over the years due to their ability to model complex nonlinear relationships and handle noisy inputs^39–42^. The architecture of MLPs, consisting of multiple layers of neurons, enables them to learn intricate patterns in data, making them suitable for modelling the nonlinear characteristics of process data in soft sensor applications^41,42^. With the advancements in artificial intelligence, various other types of neural network architectures have also been utilised in soft sensor design. Of them, LSTM neural networks and their variants have widely been employed as dynamic soft sensor models across numerous applications due to their ability to extract complex temporal dependencies in industrial process data^23,43–51^. Due to the memory units in LSTMs, they can effectively capture temporal variations in the process leading to improved predictive performance compared to static models such as the MLP neural network. Hence, both MLP and LSTM neural network architectures were incorporated and compared as the black-box component of the proposed grey-box soft sensor in this study.

The key contributions of this study can be identified as follows: A grey-box soft sensor incorporating a physics-based analytical model and a deep neural network is proposed to predict the melt viscosity of a single-screw extrusion process in real time. To the best of knowledge of the authors, this is the first study that incorporates deep learning techniques as well as a CGB model architecture to predict the melt viscosity in polymer extrusion processes. The performance of the proposed soft sensor was compared with fully-data driven models to confirm its superiority. Furthermore, its performance was compared against the radial basis function neural network-based soft sensor reported in the previous study by Deng et al.^37^ for the same task. The proposed grey-box soft sensor exhibited excellent predictive performance, outperforming the fully-data driven models as well as the soft sensor reported by Deng et al.^37^ However, it should be noted that, although the soft sensor can detect viscosity changes caused by changes in operating conditions, it cannot detect viscosity changes due to changes in material properties.

## Experimental Dataset

**Fig. 1:**
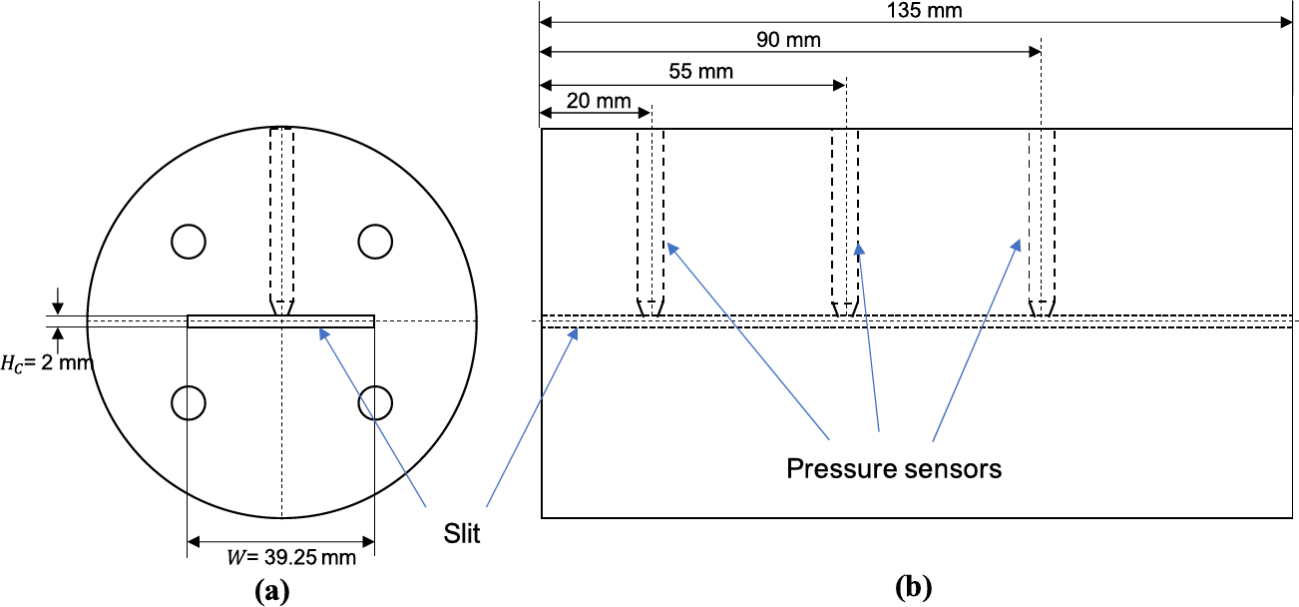
A schematic diagram of the slit die rheometer reported in the study by Deng et al.^37^: (**a**) cross-sectional view (**b**) longitudinal view.

To develop the soft sensor proposed in this study, the melt viscosity dataset reported by Deng et al.^37^ was used. In this dataset, the melt viscosity was calculated from the ratio of the shear stress to the shear rate of the melt flow. The shear stress was determined from the pressure drop along the channel of an in-line slit-die rheometer (i.e., an extruder die with a rectangular flow channel that has a large width-to-height ratio) measured in real time. A schematic diagram of the slit-die rheometer that was designed for the experiment is illustrated in Fig. 1. The shear rate was calculated from the volumetric flow rate of the melt flow through the die. The viscosity of the polymer melt can then be calculated from Eq. (1)^37^:

1 $$\:\eta\:=\frac{\tau\:}{\dot{\gamma\:}}=\frac{n{{H}_{c}}^{3}W}{4(2n+1)\dot{V}}\frac{\varDelta\:P}{L}$$

where $\:\eta\:$ denotes the melt viscosity, while $\:\tau\:$ and $\:\dot{\gamma\:}$ represent shear stress and shear rate respectively. $\:n$ is the power law index of the polymer, $\:{H}_{c}$ is the height of the channel, $\:W$ is the width of the channel, and $\:\dot{V}$ is the volumetric flow rate. $\:\varDelta\:P$ is the pressure drop along a length of $\:L$ in the channel. The volumetric flow rate ($\:\dot{V}$) was determined based on the mass throughput from the slit die and the melt density. To measure the mass throughput, the polymer melt from the slit die was collected manually at 1-min intervals and weighed. The melt density and power law index were determined using an RH7 viscometer^52^.

**Fig. 2:**
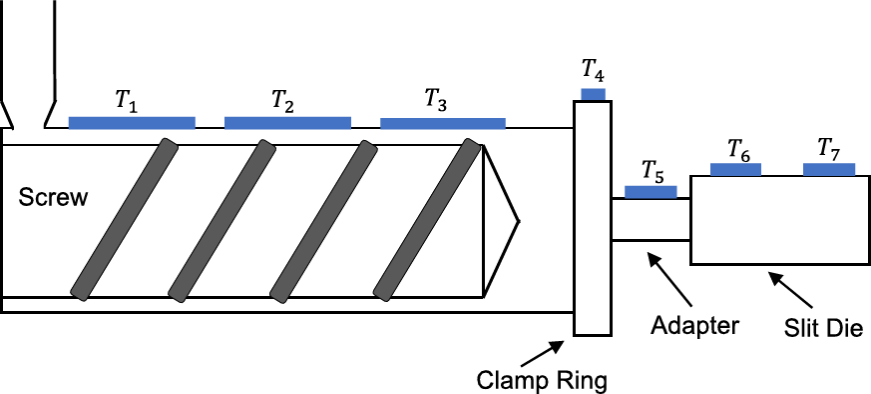
A schematic diagram indicating the heating zones of the single-screw extruder used for the experimental trial in the study by Deng et al.^37^.

**Fig. 3:**
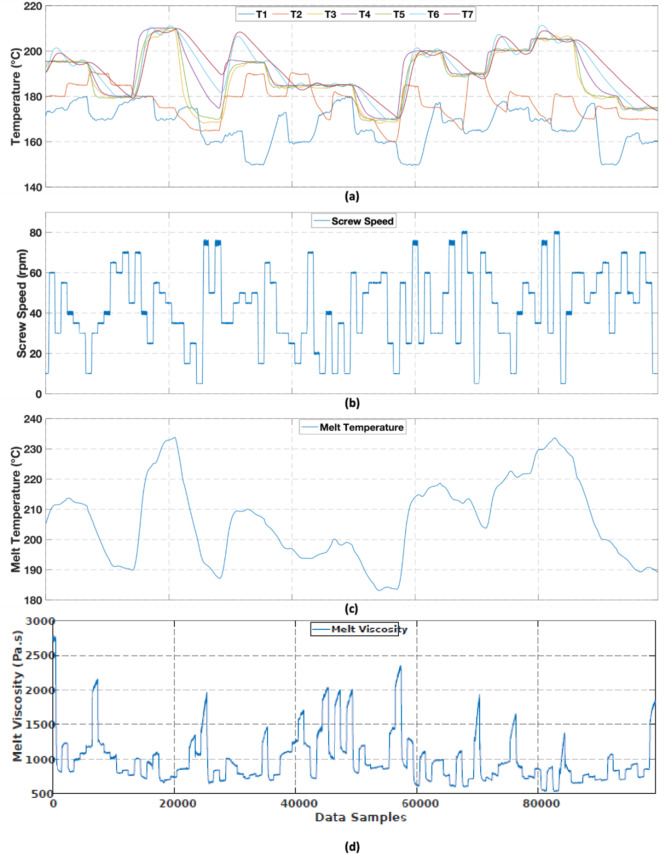
Experimental dataset: (**a**) variation of barrel set temperatures (**b**) variation of screw speed (**c**) measured melt temperature (**d**) measured melt viscosity.

The dataset was collected by conducting an experimental trial on a Killion KTS-100 single-screw extruder, using a low-density polyethylene (LDPE) material (brand name: SABIC LDPE 2102TN00W; melt flow rate: 2.5 g/10 min at 190 °C and 2.16 kg; density: 921 kgm^− 3^). The experimental trial was conducted by varying the process settings (i.e., barrel set temperatures and screw speed) of the extruder and recording the data in real time. As can be seen from Fig. 2, the extruder barrel consisted of three main heating zones (T_1_−T_3_). Four additional heating zones were also available at the clamp ring, the adapter, and the slit die (i.e., T_4_−T_7_). The barrel set temperatures and screw speed were varied using a pseudorandom sequence signal such that a wide processing range of the extruder was covered. In addition to the real-time melt viscosity data calculated from the slit die measurements, real-time measurements of barrel set temperatures (T_1_−T_7_), screw speed, and melt temperature were recorded at a sampling frequency of 10 Hz. The resulting dataset was pre-processed to eliminate melt viscosity overshoots caused by inaccurate calculation of viscosity at certain screw speed step changes. As the overshoot regions were very narrow and sparse, melt viscosity values in these regions were removed, and they were replaced using moving average smoothing. The final dataset after pre-processing consisted of a total of 99,442 data samples (see Fig. 3(a–d)).

## Preliminaries

### Grey-box model structure

**Fig. 4:**
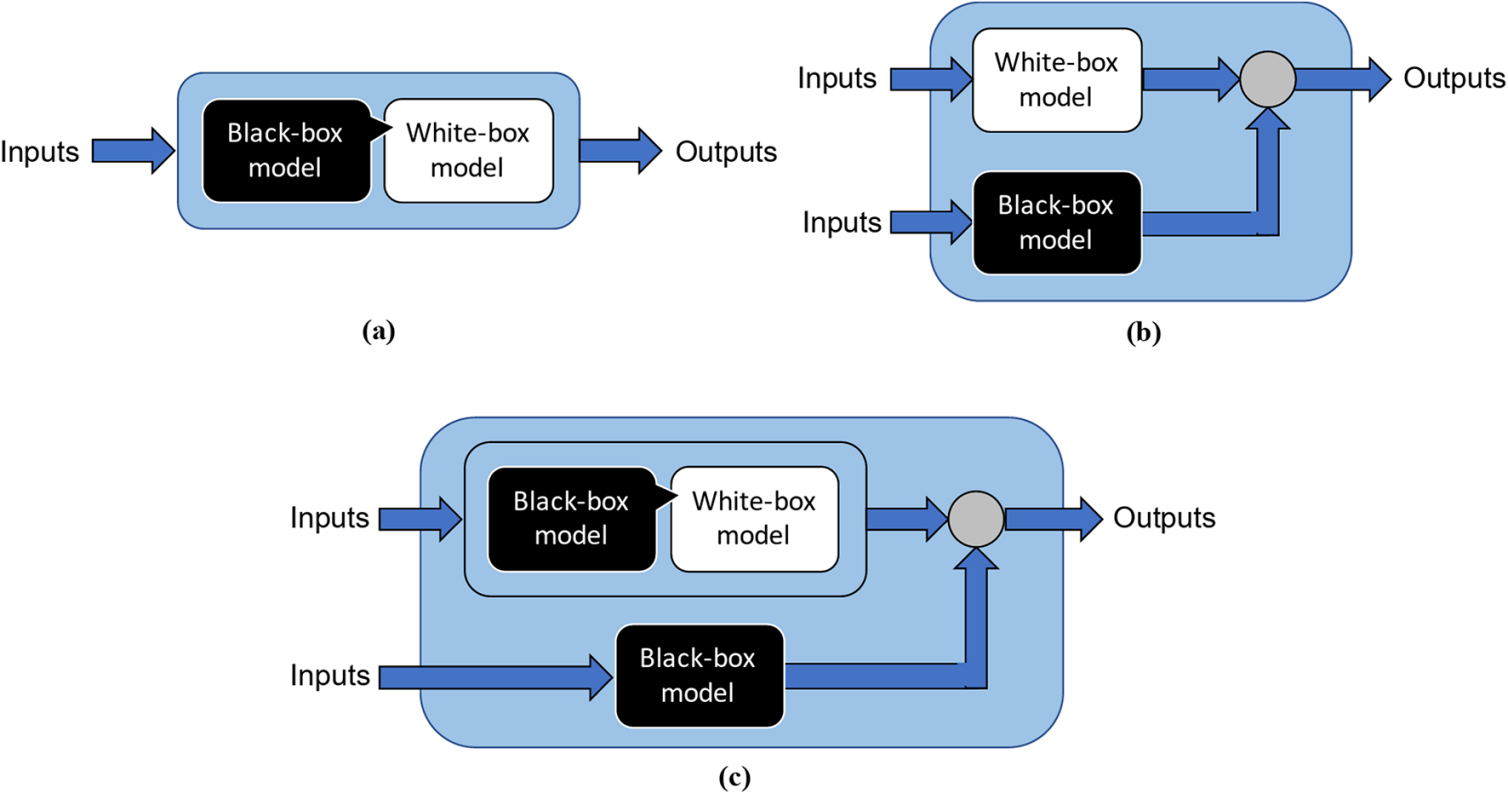
Grey-box model configurations: (**a**) serial (**b**) parallel and (**c**) combined.

In this study, a CGB model architecture^38^ as shown in Fig. 4c was used to develop the grey-box soft sensor model. This involves the integration of an SGB model component with a parallel black-box component. A CGB model architecture was chosen as it generally exhibits improved performance compared to an SGB (see Fig. 4a) or a parallel (see Fig. 4b) grey-box model configuration owing to the incorporation of both serial and parallel configurations^38^. This section summarises the main steps involved in designing the proposed CGB model.

i. Construct an SGB component to predict the target variable.
  - Develop a physics-based (i.e., white-box) model.
    2 $$\:{y}_{WB}={f}_{WB}\left({x}_{WB},\theta\:\right)$$
    Here, $\:{f}_{WB}$ denotes the physics-based model, while $\:{y}_{WB}$ is the target variable predicted by the physics-based model. $\:{x}_{WB}$ and $\:\theta\:$ represent the input variables and parameters in the physics-based model, respectively.
  - Determine the value of $\:\theta\:$ that minimises the prediction error (calculated in terms of the sum of squared errors) of the physics-based model.
    3 $$\:\widehat{\theta\:}=\underset{\theta\:}{\text{arg\:min}}\sum\:_{i=1}^{M}{\left({y}_{WB,i}-\:{y}_{m,i}\right)}^{2}$$
    where $\:{y}_{m,i}$ is the $\:i$^th^ measured value of the target variable, $\:{y}_{WB,i}$ is the $\:i$^th^ prediction by the physics-based model, and $\:M$ is the number of training data points.
  - Obtain the SGB component by combining the optimised parameters$\:\:\widehat{\theta\:}$ with the physics-based model.
    4 $$\:{y}_{SGB}={f}_{WB}\left({x}_{WB},\widehat{\theta\:}\right)$$
    where $\:{y}_{SGB}$ is the target variable predicted by the SGB component.

ii. Construct a data-driven (i.e., black-box) component to predict the prediction error of the SGB component.
  - Calculate the prediction error of the SGB component.
  5 $$\:{e}_{SGB}={y}_{m}-{y}_{SGB}$$
  where $\:{e}_{SGB}$ is the vector that contains the prediction errors of the SGB component calculated as the difference between the experimentally measured target values ($\:{y}_{m}$) and the SGB model predictions ($\:{y}_{SGB}).$
b. Develop the parallel black-box component.
  6 $$\:{\widehat{e}}_{SGB}={f}_{BB}\left({x}_{BB},\varnothing\:\right)$$
  where $\:{\widehat{e}}_{SGB}$ is the prediction by the parallel black-box component. $\:{f}_{BB}$ is the complex nonlinear function of the black-box component, while $\:{x}_{BB}$ and $\:\varnothing\:$ denote the input features and parameters of the black-box component respectively.
c. Determine the value of $\:\varnothing\:$ that minimises the prediction error (calculated in terms of the sum of squared errors) of the black-box component.
  7 $$\:\widehat{\varnothing\:}=\underset{\varnothing\:}{\text{arg\:min}}\sum\:_{i=1}^{M}{\left({\widehat{e}}_{SGB,i}-\:{e}_{SGB,i}\right)}^{2}$$
  where $\:{e}_{SGB,i}$ is the $\:i$^th^ calculated prediction error of the SGB component, $\:{\widehat{e}}_{SGB,i}$ is the $\:i$^th^ prediction by the black-box component, and $\:M$ is the number of training data points.
d. Obtain the black-box model predictions ($\:{y}_{BB}$) with the optimised parameters$\:\:\widehat{\varnothing\:}$
  8 $$\:{y}_{BB}={f}_{BB}\left({x}_{BB},\widehat{\varnothing\:}\right).$$

iii. Construct the CGB model by combining the SGB component with the parallel black-box component.
  9 $$\:{y}_{CGB}={y}_{SGB}+{y}_{BB}$$
  where, $\:{y}_{CGB}$ is the final prediction of the CGB model.

To construct the parallel black-box component (represented by $\:{f}_{BB}$ in Eq. (8) of the grey-box soft sensor model, neural networks with two different architectures were used. A deep neural network with an MLP architecture and a deep LSTM neural network were employed.

### MLP neural network

**Fig. 5:**
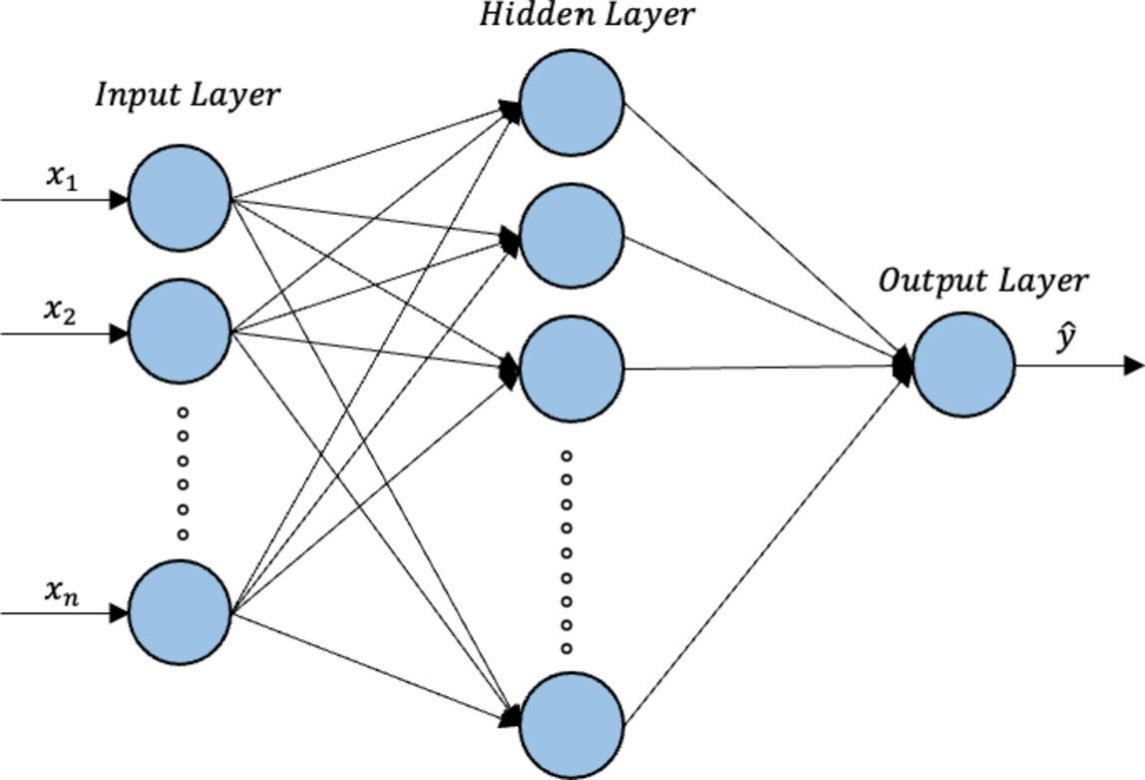
MLP neural network architecture.

An MLP neural network is a feedforward neural network. A perceptron is a single neuron, which is a computational unit that processes a set of weighted inputs using an activation function to produce an output. In an MLP neural network, such neurons are stacked to form a hidden layer, and MLP neural networks are composed of one or more such hidden layers. Deep networks can be constructed by stacking multiple hidden layers. Figure 5 illustrates the network architecture of an MLP with an input layer, one hidden layer, and an output layer.

The number of neurons in the input and output layers is determined by the number of input and target variables in the problem under consideration. The number of neurons in a hidden layer and the number of hidden layers in the final MLP neural network model are usually determined by a trial-and-error approach, such that the maximum predictive performance of the model is achieved. The input-output relationship of an MLP neural network with a single hidden layer can be described by Eq. (10).

10 $$\:\widehat{y}=g\left(\sum\:_{j=1}^{p}{w}_{j}^{\left(2\right)}f\left(\left(\sum\:_{i=1}^{q}{w}_{ij}^{\left(1\right)}{x}_{i}\right)+{b}_{j}^{\left(1\right)}\right)+{b}^{\left(2\right)}\right)$$

The input vector $\:x$ contains $\:q$ input variables, and this input vector is combined with the weights vector $\:{w}^{\left(1\right)}.$ The weight $\:{w}_{ij}^{\left(1\right)}$ corresponds to the connection between the $\:i$^th^ input and the $\:j$^th^ neuron of the hidden layer. The hidden layer consists of $\:p$ hidden units. The weighted sum calculated at each neuron along with the corresponding bias value $\:{b}_{j}^{\left(1\right)}$ is then subjected to an activation function $\:f$. The resulting vector and the bias value $\:{b}^{\left(2\right)}$ are then combined with the weights vector $\:{w}^{\left(2\right)}$ corresponding to the output layer and subjected to an activation function $\:g$ to obtain the predicted output $\:\widehat{y}$. The activation functions could be any arbitrary function including the sigmoid, hyperbolic tangent (tanh), or rectified linear unit (ReLU) functions. Equation (10) can be extended to accommodate more hidden layers to represent a deep network.

Neural network training consists of two main phases: forward propagation and backpropagation. During forward propagation, the input features are combined with the weights and biases, and the network makes a prediction based on learned features using the activation functions. After each iteration of the forward propagation, the prediction error is calculated by taking the square of the difference between the actual and predicted values. The prediction errors are averaged over the entire training data using a cost function. The mean square error (MSE) shown in Eq. (11) is generally chosen as the cost function, where $\:{m}_{t}$ denotes the number of training samples.

11 $$\:MSE=\:\frac{1}{{m}_{t}}\sum\:_{i=1}^{{m}_{t}}{({y}_{i}-{\widehat{y}}_{i})}^{2}$$

Forward propagation is followed by backpropagation, during which the gradient of the loss function with respect to the weights is calculated. Backpropagation is carried out using an optimisation algorithm such as the gradient descent to find the weights and biases that minimise the cost function in Eq. (11). The full mathematical derivation is not presented here but can be found in the literature^53^.

### LSTM neural network

**Fig. 6:**
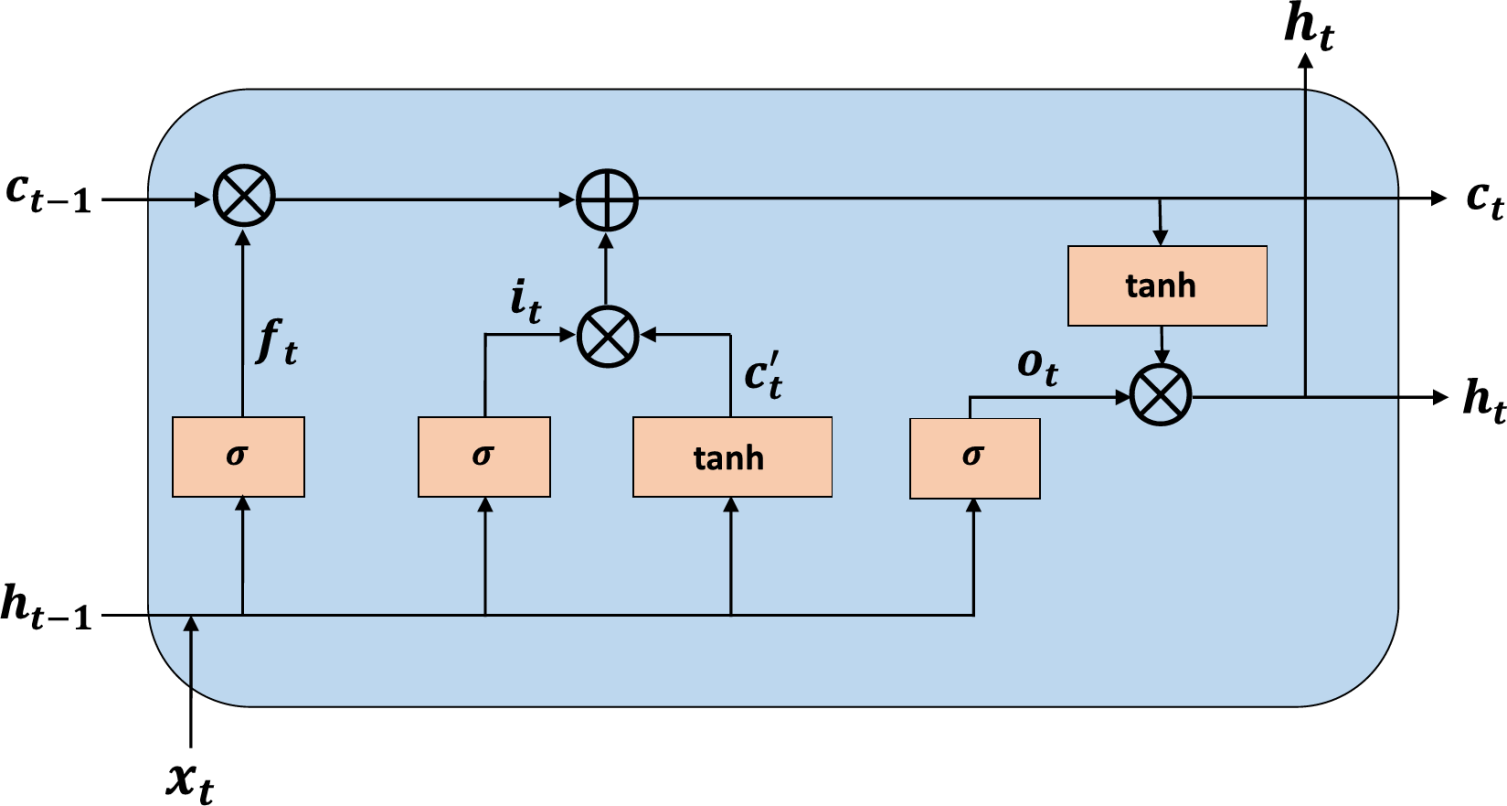
The structure of an LSTM cell.

The LSTM neural network is a variant of the recurrent neural network (RNN), which was designed to overcome the issues of gradient vanishing and gradient exploding present in RNNs. LSTM networks consist of three gates; namely, the input, forget, and output gates, which enable the handling of long-term dependencies in the data. The structure of an LSTM cell is illustrated in Fig. 6. The internal mechanisms of an LSTM cell can be presented as shown in Eqs. (12), (13), (14), (15), (16), (17):

12 $$\:{f}_{t}=\sigma\:({W}_{f}.\left[{h}_{t-1},{x}_{t}\right]+{b}_{f})$$

13 $$\:{i}_{t}=\:\sigma\:({W}_{i}.\left[{h}_{t-1},{x}_{t}\right]+{b}_{i})$$

14 $$\:{c}_{t}^{{\prime\:}}=\text{t}\text{a}\text{n}\text{h}({W}_{c}.\left[{h}_{t-1},{x}_{t}\right]+{b}_{c})$$

15 $$\:{c}_{t}={f}_{t}*{c}_{t-1}+{i}_{t}*{c}_{t}^{{\prime\:}}$$

16 $$\:{o}_{t}=\sigma\:({W}_{o}\left[{h}_{t-1},{x}_{t}\right]+{b}_{o)}$$

17 $$\:{h}_{t}={o}_{t}*\text{t}\text{a}\text{n}\text{h}\left({c}_{t}\right)$$

Here, $\:{x}_{t}$ is the input matrix. $\:{f}_{t}$, $\:{i}_{t}$, and $\:{o}_{t}$ represent the forget, input, and output gates, respectively. $\:{c}_{t}$ indicates the current cell state and $\:{c}_{t}^{{\prime\:}}$ indicates the vector of new data to be added to the cell state. $\:{h}_{t}$ is the hidden state of the LSTM cell. $\:{W}_{f}$, $\:{W}_{i}$, $\:{W}_{c}$, and $\:{W}_{o}$ denote the corresponding weight matrices, while $\:{b}_{f}$, $\:{b}_{i}$, $\:{b}_{c}$, and $\:{b}_{o}$ represent the corresponding bias terms. $\:\sigma\:$ represents the activation function.

## Soft Sensor Development

In this study, two grey-box soft sensor models were constructed. The only difference between the two models is in the type of neural network used as the black-box component. Model A incorporated an LSTM neural network as the black-box component, while Model B used an MLP neural network. Alongside these grey-box models, two fully data-driven models were also constructed: Model C, based on an LSTM neural network, and Model D, based on an MLP neural network. The fully data-driven models were designed to serve as benchmarks for evaluating the grey-box models. The purpose of this comparison was to determine whether integrating physics-based knowledge into soft sensor design improves the predictive performance of the soft sensor. Table 1 provides a description of the different models developed in this study.

**Table 1:** Grey-box and data-driven soft sensor models developed in this study.

Model name	Model type	Description
Model A	Grey box	CGB with an LSTM neural network as the black-box component
Model B	Grey box	CGB with an MLP neural network as the black-box component
Model C	Data driven	LSTM neural network
Model D	Data driven	MLP neural network

### Grey-box soft sensor models

This section discusses the development of the grey-box soft sensor Models A and B. First, the construction of the SGB component is discussed followed by the integration of the parallel black-box component.

Polymer melts are pseudo-plastic fluids, where the melt viscosity decreases with increasing shear rates^54^. Generally, the shear rates generated during polymer extrusion processes are within the range of 1−10^4^ s^−1^. Melt viscosities within this region can be reasonably approximated using the power law of Ostwald and de Waele^55,56^. This power law equation is shown in Eq. (18):

18 $$\:\eta\:=m{\dot{\gamma\:}}^{n-1}$$

Here, $\:\eta\:$ is the melt viscosity at a shear rate of $\:\dot{\gamma\:}$. $\:m$ is the melt consistency index, while $\:n$ denotes the power law index. The power law index varies between 0 and 1 for pseudo-plastic fluids such as polymer melts. The melt consistency index is a temperature-dependent parameter, and the melt consistency index at temperature $\:T$ denoted by $\:m\left(T\right)$ can be calculated from Eq. (19)^57^:

19 $$\:m\left(T\right)={m}_{r}{e}^{{\alpha\:}_{T}({T}_{r}-T)}$$

where $\:{m}_{r}$ is the reference melt consistency index at the reference temperature $\:{T}_{r}$, while $\:{\alpha\:}_{T}$ represents the temperature coefficient.

In this study, the power law equation in Eq. (19) was used for constructing the physics-based model. For single-screw polymer extruders, the shear rate in the screw channel of the melt conveying zone (i.e., the final zone of the processing screw) can be approximated from Eq. (20) using the flat plate approximation model^54^.

20 $$\:\dot{\gamma\:}\approx\:\frac{\pi\:DN}{H}$$

where $\:D$,$\:\:N$, and $\:H$ represent the screw diameter, screw rotational speed, and the depth of the screw channel, respectively. By substituting Eqs. (19), (20) in Eq. (18), the following expression can be obtained for calculating the melt viscosity.

21 $$\:\eta\:={m}_{r}{e}^{{\alpha\:}_{T}({T}_{r}-T)}{\left(\frac{\pi\:DN}{H}\right)}^{n-1}$$

The parameters $\:D$ and $\:H$ are geometrical parameters of the extruder, while $\:N$ is a processing parameter, all of which are readily available to the machine operators. However, material-related properties such as $\:{m}_{r}$,$\:\:{T}_{r}$,$\:\:{\alpha\:}_{T}$, and $\:n$ are not readily available to the machine operators and these values may not be available in the material datasheets as well. As a result, these parameters would have to be determined via offline experimentation. Hence, it would not be practical to use the physics-based model in Eq. (21) in its current form, to predict the melt viscosity in real time during polymer extrusion processes in an industrial setting. Therefore, it would be more practical to optimise these unknown parameters using actual experimental data. To make this possible, the model presented in Eq. (21) can be re-arranged as follows:

22 $$\:\eta\:={m}_{r}{e}^{{\alpha\:}_{T}({T}_{r}-T)}{\left(\dot{\gamma\:}\right)}^{n-1}$$

By taking the natural logarithm on both sides of Eq. (22), the model can be transformed into Eq. (23):

23 $$\:\text{ln}\left(\eta\:\right)=\text{ln}\left({m}_{r}\right)+{\alpha\:}_{T}\left({T}_{r}-T\right)+(n-1)\text{ln}\left(\dot{\gamma\:}\right)$$

24 $$\:\text{ln}\left(\eta\:\right)=\left(n-1\right)\text{ln}\left(\dot{\gamma\:}\right)-{\alpha\:}_{T}T+\text{ln}\left({m}_{r}\right)+{\alpha\:}_{T}{T}_{r}$$

Equation (24) has the form, $\:y=\:{\theta\:}_{1}{u}_{1}+{\theta\:}_{2}{u}_{2}{+\theta\:}_{3}$, where

25 $$\:y=\text{ln}\left(\eta\:\right)$$

26 $$\:{u}_{1}=\text{ln}\left(\dot{\gamma\:}\right)$$

27 $$\:{u}_{2}=T$$

28 $$\:{\theta\:}_{1}=n-1$$

29 $$\:{\theta\:}_{2}=-{\alpha\:}_{T}$$

30 $$\:{\theta\:}_{3}=\text{ln}\left({m}_{r}\right)+{\alpha\:}_{T}{T}_{r}$$

Here, $\:{u}_{1}$ and $\:{u}_{2}$ constitute the input vector $\:{x}_{WB}$, while $\:{\theta\:}_{1}$,$\:\:{\theta\:}_{2}$, and $\:{\theta\:}_{3}$ constitute the parameter vector $\:\theta\:$ of the physics-based model as indicated by Eq. (2). $\:{u}_{1}$ was computed from Eqs. (20) and (26) for all data samples collected during the experimental trial. $\:{u}_{2}$ denotes the melt temperature ($\:T$), and the bulk melt temperature measured using a wall-mounted thermocouple at the adapter of the extruder during the experimental trial was used (see Fig. 3c). The geometrical parameters of the extruder required to calculate the shear rate as shown in Eq. (21) (i.e., screw diameter (D) and screw channel depth (H)) were obtained from the single-screw extruder used for the experimental trial. The values of D and H were found to be 25 mm and 1.43 mm respectively. The unknown parameter vector $\:\theta\:$ was optimised using the training set. Both linear regression and particle swarm optimisation (PSO) were used to optimise $\:\theta\:$ and the performance of the two algorithms are compared in the ‘Results and Discussion’ section. Then, the optimised parameter vector $\:\widehat{\theta\:}$ was combined with the physics-based model to obtain the optimised model in Eq. (31):

31 $$\:\text{ln}\left(\eta\:\right)={\widehat{\theta\:}}_{1}\text{ln}\left(\dot{\gamma\:}\right)-{\widehat{\theta\:}}_{2}T+{\widehat{\theta\:}}_{3}$$

Then, the final SGB component in Eq. (32) was obtained by removing the logarithmic transformation.

32 $$\:\eta\:={e}^{\left({\widehat{\theta\:}}_{1}\text{ln}\left(\dot{\gamma\:}\right)-{\widehat{\theta\:}}_{2}T+{\widehat{\theta\:}}_{3}\right)}$$

Next, the fine-tuned SGB component (in Eq. (32)) was used to make melt viscosity predictions for the training set. This was followed by the calculation of the prediction errors of the SGB component over the entire training set using Eq. (5). These calculated prediction errors were subsequently used as the target values to train the parallel black-box component.

As shown in Table 1, LSTM and MLP neural networks were used as the parallel black-box component for Models A and B, respectively. For both LSTM and MLP black-box components, eight features (i.e., seven barrel set temperatures and screw speed) were used as inputs to predict the output (i.e., prediction error of the SGB model). These input features were chosen as they are the primary process control variables in polymer extruders, and these parameters are known to have a significant influence on the melt viscosity^2,6,33–36^. Equations (33), (34) describe the inputs and outputs used for the MLP black-box component of Model B.

33 $$\:{y}_{t}={g}_{MLP}\left({x}_{t}\right)$$

34 $$\:{x}_{t}=\{{T}_{1}\left(t\right),{T}_{2}\left(t\right),{T}_{3}\left(t\right),{T}_{4}\left(t\right),{T}_{5}\left(t\right),{T}_{6}\left(t\right),{T}_{7}\left(t\right),\omega\:(t\left)\right\}$$

where $\:{x}_{t}$ is the value of the input feature vector at time $\:t$, while $\:{y}_{t}$ is the value of the target variable at time $\:t$. $\:{T}_{i}\left(t\right){|}_{i=\text{1,2},\dots\:.,7}$ denote the barrel set temperatures at time $\:t$, and $\:\omega\:\left(t\right)$ represents the screw speed at time $\:t$. $\:{g}_{MLP}$ is the nonlinear function learned by the MLP neural network.

To construct the black-box component of Model A, considering the dynamic nature of LSTM neural networks, past values of the input features were also used in addition to the present input values as shown in Eq. (35):

35 $$\:{y}_{t}={g}_{LSTM}({x}_{t},{x}_{t-1},\dots\:,{x}_{t-d})$$

where $\:d$ is the number of past time steps or the width of the sliding window of the LSTM network, and$\:\:{g}_{LSTM}$ is the nonlinear function learned by the LSTM neural network.

Before training the LSTM black-box component of Model A, the entire dataset was split into train, validation, and test sets. Random splitting was used to ensure the same distribution of data across the three sets. However, when dealing with LSTM networks, it is necessary to maintain the temporal order of the dataset and random splitting disrupts the temporal order which may result in data leakage. Therefore, to prevent this, the entire dataset was serialised using a sliding window with a suitable width before feeding the network. Serialising the dataset before splitting it randomly into train, validation, and test sets could ensure that the temporal order of the data is maintained^58^. To prevent data leakage, non-overlapping sample sequences were created during the serialisation process. Using non-overlapping samples ensures that no sequence samples in the serialised dataset have exact duplicates in the train, validation, and test sets. This ensures that the model does not peak into future time steps in addition to past time steps and hence prevents data leakage which could cause the model to produce overly optimistic results.

**Fig. 7:**
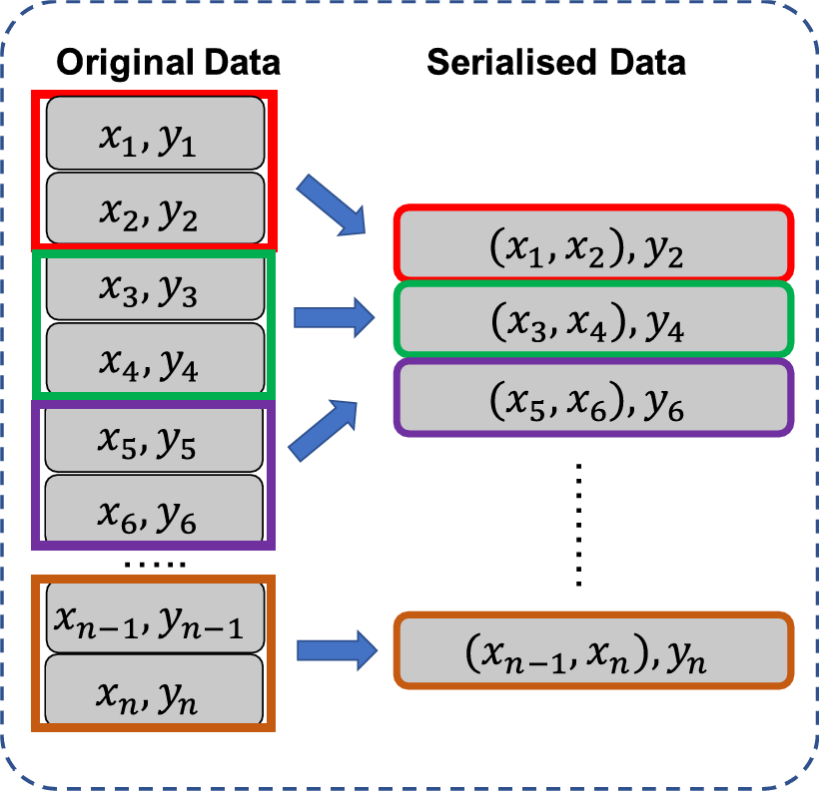
Serialisation of the dataset.

Consider the original dataset $\:\left\{X,Y\right\}=\left\{\left({x}_{i},{y}_{i}\right){|}_{i=1,\:2,\dots\:,k}\right\}$, where $\:{x}_{i}$ and $\:{y}_{i}$ denote the values of the input features and the target variable corresponding to the $\:i$^th^ data sample, and $\:k$ is the total number of data samples. A sliding window with a width of $\:d$ can then be used to scan and serialise the dataset. This results in a serialised dataset $\:\left\{{X}^{*},{Y}^{*}\right\}=\left\{\left({x}_{j}^{*},{y}_{j}^{*}\right){|}_{j=1,\:2,\dots\:,k-d+1}\right\}$, where $\:{x}_{j}^{*}=\left\{{x}_{j-d+1},\dots\:,{x}_{j-1},{x}_{j}\right\}$ and $\:{y}_{j}^{*}=\left\{{y}_{j}\right\}$. An example of this serialisation process for $\:d=2$ is illustrated in Fig. 7.

The dataset used to train and test the soft sensor models consisted of a total of 99,442 data points. Before splitting, it was serialised using a suitable window size as described above. The size of the sliding window was fine-tuned along with other hyperparameters during training, and the optimum size was found to be 2. Hence, the serialisation resulted in a total of 49,721 sample sequences. They were split into train, validation, and test sets at a ratio of 60:20:20. This resulted in 29,832, 9,944, and 9,945 sample sequences in the train, validation, and test sets, respectively. The same train, validation, and test splits were used to train the black-box components of both Models A and B.

The black-box components of Models A and B were trained on the training set, while the hyperparameters were optimised using the grid searching technique based on the models’ performance on the validation set. Although there are several hyperparameter tuning techniques such as gradient-based optimisation, Bayesian optimisation, and Metaheuristic algorithms, grid searching (which is a model-free algorithm) was used due to its simplicity and exhaustive search provided^59^. The test set was used to assess the model’s performance on new unseen data. Under hyperparameter tuning, the number of hidden layers, number of neurons per hidden layer, batch size, and the number of training iterations were fine tuned as both MLP and LSTM networks are highly sensitive to these hyperparameters. For the LSTM network, the width of the time window was also treated as an additional hyperparameter.

**Fig. 8:**
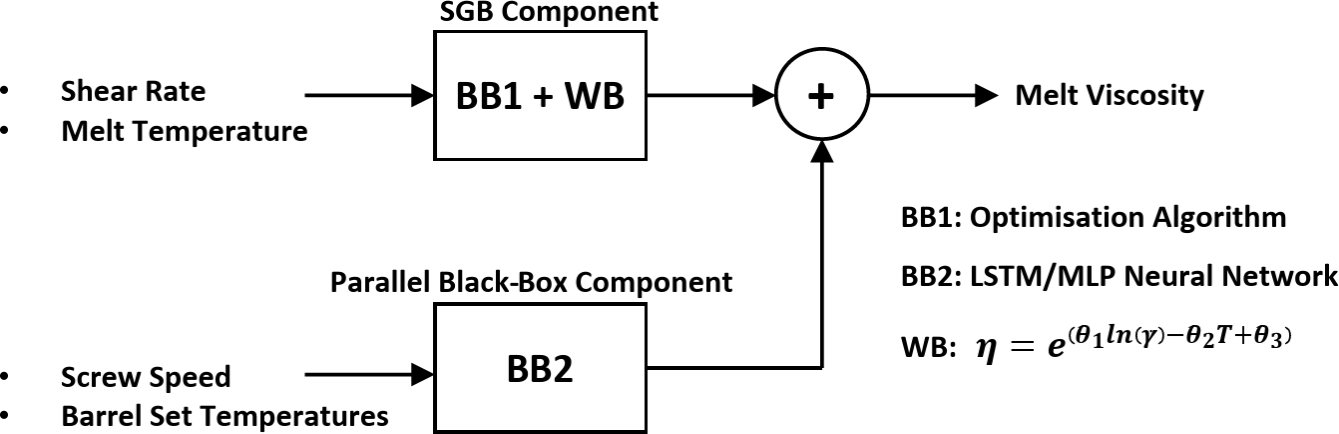
Proposed CGB model. BB and WB denote black-box and white-box (i.e., physics-based) model components respectively.

Figure 8 illustrates the final CGB model. The SGB component takes in shear rate and melt temperature as inputs and estimates the melt viscosity using the parameters optimised with an optimisation algorithm. Simultaneously, the black-box component takes the barrel set temperatures and screw speed as model inputs and estimates the prediction error of the SGB component. Here, different inputs were used for the black-box component compared to the SGB component. This was done to incorporate the control variables (i.e., barrel set temperatures and screw speed) of the extruder as model inputs. However, it should be noted that the shear rate and melt temperature which were used as inputs to the SGB component are also functions of the screw speed and barrel set temperatures.

Finally, the black-box model prediction is added to the SGB prediction to obtain the final melt viscosity prediction of the CGB model. The generalisation performance of the CGB model was further evaluated using the unseen test set. The performance of the CGB model is discussed in detail in the ‘Results and Discussion’ section.

### Data-driven soft sensor models

The same train, validation, and test split used for the grey-box models were used to design the fully data-driven Models C and D shown in Table 1. The same input features (i.e., seven barrel set temperatures and screw speed) used for the parallel black-box component of the grey-box models were used for Models C and D as well. The melt viscosity was used as the output variable, as these models were designed to predict the melt viscosity directly. Similar to the black-box components of the grey-box models, the hyperparameters of the fully data-driven models were also fine-tuned using the grid searching technique.

### Performance evaluation metrics

The soft sensor models proposed in this study were trained on a computer with an Apple M1 chip and 8GB RAM with the TensorFlow 2.15.0 backend on Python 3.11.8. The accuracy of the trained soft sensor models was evaluated using the root mean square error (RMSE), normalised RMSE (NRMSE), and root mean square percentage error (RMSPE) error metrics which are defined in Eqs. (36), (37), (38), respectively. Figure 3d shows that the melt viscosity values of the collected dataset are in the range 500–3000. Therefore, it may be difficult to get an insight into the accuracy of the models merely based on the RMSE metric due to the wide range of melt viscosity values observed in the dataset. Hence, the NRMSE was used to better interpret the RMSE values by eliminating the effect of the large value range of the melt viscosity values. The RMSPE was used to enable comparison of the results of this study with those of the previous study by Deng et al.^37^.

36 $$\:RMSE=\sqrt{\sum\:_{i=1}^{{N}_{s}}\frac{{({y}_{i}-{\widehat{y}}_{i})}^{2}}{{N}_{s}}}$$

37 $$\:NRMSE=\:\frac{RMSE}{{y}_{max}-{y}_{min}}$$

38 $$\:RMSPE=\sqrt{\frac{1}{{N}_{s}}\sum\:_{i=1}^{{N}_{s}}{\left(\frac{{y}_{i}-{\widehat{y}}_{i}}{{y}_{i}}\right)}^{2}}\times100\%$$

$\:{y}_{i}$ and $\:{\widehat{y}}_{i}$ denote the $\:i$^th^ measured and predicted outputs respectively, while $\:{N}_{s}$ denotes the number of data points. $\:{y}_{max}$ and $\:{y}_{min}$ denote the maximum and minimum values in the measured output respectively.

In addition to the above error metrics, the Kling-Gupta Efficiency (KGE) of the predictions made by the soft sensor models was also calculated in order to further evaluate the performance of the models. The KGE introduced by Gupta et al.^60^ provides a more balanced evaluation of the performance of the models by decomposing the efficiency into three components: correlation, bias, and variability. The KGE is calculated as shown in Eq. (39):

39 $$\:KGE=1-\sqrt{{\left(r-1\right)}^{2}+{\left(\alpha\:-1\right)}^{2}+{\left(\beta\:-1\right)}^{2}}$$

where $\:r$ denotes the correlation coefficient, $\:\alpha\:$ is the bias ratio (i.e., ratio of means between the predicted and measured values), and $\:\beta\:$ represents the variability ratio (i.e., ratio of standard deviations between the predicted and measured values).

## Results and Discussion

This section provides an in-depth analysis of the performance of the grey-box soft sensor models proposed in this study. As discussed in the ‘Soft Sensor Development’ section, both linear regression and PSO algorithms were used to optimise the parameter vector $\:\theta\:$ of the physics-based model in the SGB component of the grey-box soft sensor models. The performance of the SGB component on the train, validation, and test sets when optimised with each algorithm are presented in Table 2. For the PSO algorithm, the number of particles in the swarm and the maximum number of iterations were set to 100 and 200, respectively.

**Table 2:** Performance comparison of the SGB component optimised with linear regression and PSO algorithms.

Optimisation algorithm	Model performance			Optimised model parameters		
	Train RMSE	Validation RMSE	Test RMSE	$\:\widehat{{\theta\:}_{1}}$	$\:\widehat{{\theta\:}_{2}}$	$\:\widehat{{\theta\:}_{3}}$
Linear regression	167.2750	172.2734	169.6063	-0.4394	-0.0065	11.5289
PSO	167.2703	172.2678	169.6020	-0.4351	-0.0065	11.5077

**Fig. 9:**
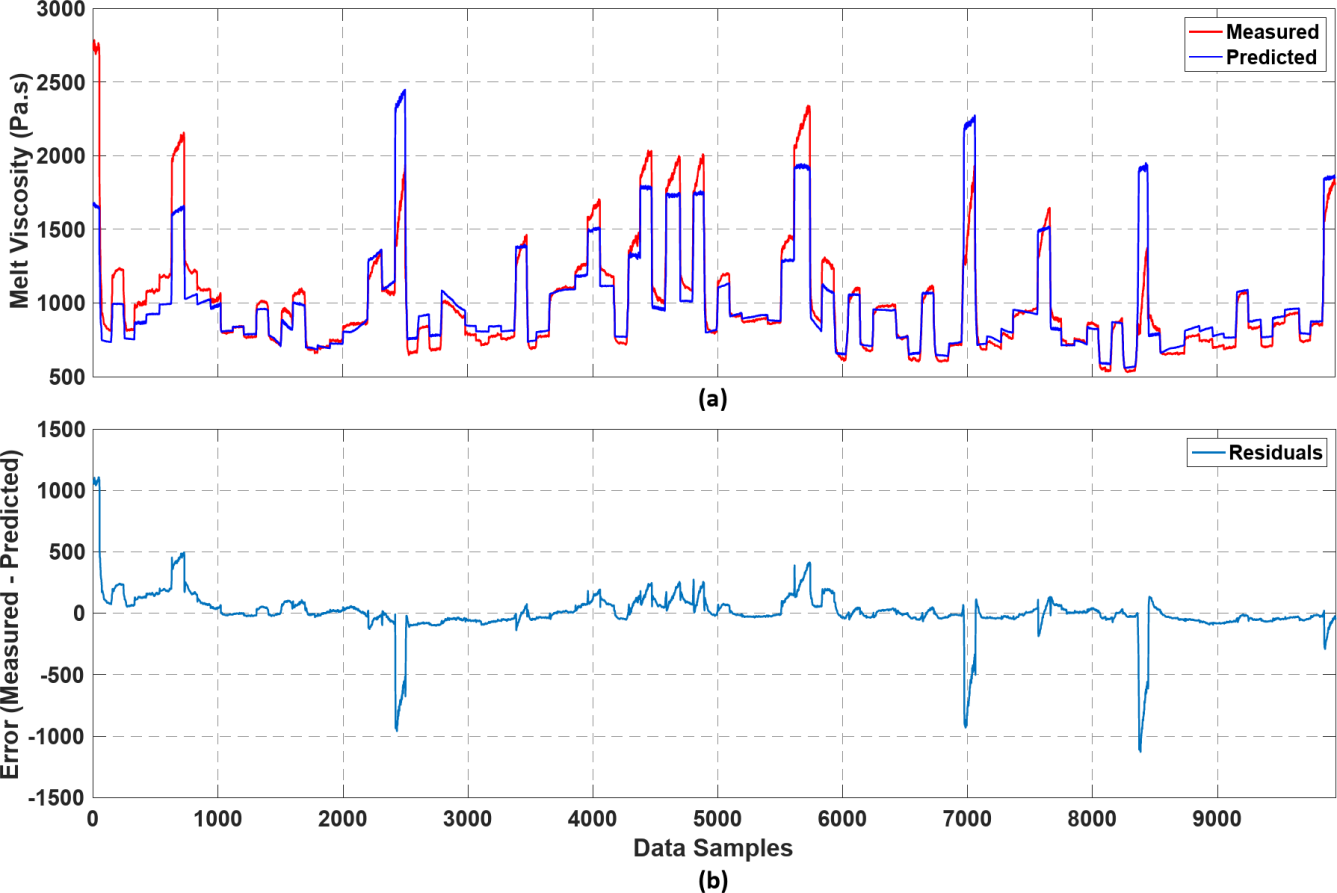
Performance of the SGB component of the CGB model: (**a**) comparison of SGB model predictions with experimentally measured melt viscosity values in the test set (**b**) residual plot for the SGB component.

It is clear from the results presented in Table 2 that there is no significant influence on the predictive performance of the SGB component of the grey-box model by the optimisation algorithm used. Hence, the linear regression algorithm was chosen considering its simplicity. The similar RMSE values exhibited on the train, validation, and test sets suggest that the model can generalise well on unseen data without overfitting. The fine-tuned parameters of the SGB component were found to be −0.4394, −0.0065, and 11.5289 for $\:{\widehat{\theta\:}}_{1}$,$\:\:{\widehat{\theta\:}}_{2}$, and $\:{\widehat{\theta\:}}_{3}$, respectively, using linear regression. Figure 9a illustrates a comparison of the SGB model predictions with the experimentally measured melt viscosity values on the unseen test set, while Fig. 9b shows the residual plot that indicates the error between the predicted and measured melt viscosity values across the test set.

Figure 9a shows that the predictions of the SGB component follow the dynamics in the data well but exhibit significant deviations from the experimentally measured melt viscosity values. This is further evident from the prediction errors with magnitudes in excess of 500 indicated by the residual plot in Fig. 9b. Since the melt viscosity is a function of shear rate, which in turn is a function of screw speed, melt viscosity values predicted by the SGB component can follow the changes in screw speed quite well. However, the significant deviations between the SGB predictions and experimentally measured values can be attributed to several sources of error. As discussed in the ‘Soft Sensor Development’ section, the unknown parameters in the SGB component are functions of material-related parameters (i.e., melt consistency index, temperature coefficient, and power law index). These parameters were fine-tuned using linear regression such that the SGB component fits the experimental data. Hence, these fine-tuned parameters could slightly vary from the actual values of the polymeric material. Moreover, the power law index is a temperature-dependent parameter, but it was defined as a constant parameter in the SGB component. Furthermore, the melt viscosity may vary as the melt flows from the screw channel to the die and the melt viscosity measured at the die could be different from the melt viscosity at the melt conveying zone of the extruder that is predicted by the SGB component. All these causes may adversely affect the predictive performance of the SGB component. Hence, the parallel black-box component was used to predict the errors of the SGB component to obtain melt viscosity predictions with better accuracy.

After obtaining the fine-tuned SGB component, the parallel black-box component was then trained to predict the residual between the SGB predictions and the experimentally measured melt viscosity values. The black-box component was trained on the training set while its hyperparameters were fine-tuned based on its performance on the validation set. The predictions from the black-box component were then added to the predictions from the SGB component, to get the final melt viscosity predictions of the CGB model. Finally, the fine-tuned CGB model was evaluated on the test set. Model A reported RMSE values of 4.4072, 4.9061, and 5.1743 on the training, validation, and test sets, respectively. The respective RMSE values for Model B was found to be 4.3671, 5.0219, and 5.0394.

These results show that both Models A and B have shown slightly higher RMSE values on the validation and test sets compared to the training set. However, the validation and test RMSE values are quite similar indicating good generalisation performance on unseen data. Furthermore, the final CGB models show a significant improvement in performance compared to the SGB component, and this indicates that the black-box components of both Models A and B have substantially contributed to compensating for the prediction errors of the SGB component. Although both Models A and B exhibit good predictive performance, Model B has slightly better performance compared to Model A with a reduction of 2.6% in the RMSE value on unseen test data. These findings are interesting, as one would expect Model A to outperform Model B due to the use of an LSTM neural network as the black-box component in Model A, which can extract temporal features in the data, unlike the MLP neural network that was used as the black-box component in model B. This behaviour may be attributed to the following: According to Fig. 3, the dataset used in this study was collected by varying the extruder process parameters using a pseudorandom sequence signal, which resulted in frequent step changes in the extruder barrel set temperatures and screw speed. Figure 3(b, d) show that the resulting melt viscosity is highly sensitive to the screw speed step changes and has immediately responded to these changes without any noticeable delays. Moreover, as can be observed from Fig. 9a, the SGB component seems to follow the dynamics in the data despite its static model structure, and this might have left limited room for the LSTM (which models the SGB residuals) to contribute additional value. Hence, there may not have been any temporal features that the LSTM neural network could learn in addition to what the MLP neural network, which does not have a memory component, could learn. Additionally, the width of the sliding window used to serialise the dataset was also fine-tuned as a hyperparameter, and the best value was found to be 2. Any further increments resulted in a reduction in predictive performance of Model A. Therefore, in this case, it is clear that the CGB models exhibit comparable predictive performance regardless of whether an MLP or LSTM neural network is employed as the parallel black-box component, with the CGB model with an MLP neural network having a slight edge in performance.

Next, the performance of the CGB models were compared with the fully data-driven models. The hyperparameters of all the fine-tuned models are provided in Table 3. The performance of the CGB Models A and B as well as the data-driven Models C and D on the test set are summarised in Table 4. In addition to these models, the performance of the RBF neural network-based soft sensor model proposed in the work by Deng et al.^37^ (i.e., Model E) is also provided in Table 4 for comparison.

**Table 3:** Fine-tuned hyperparameters of all models developed in this study.

Model	Model A	Model B	Model C	Model D
No. of hidden layers	5	7	4	5
No. of neurons per hidden layer	200	250	180	200
Batch size	64	256	64	128
Training iterations	5623	5597	4217	5438
Width of the sliding window	2	N/A	2	N/A
Optimiser	Adam	Adam	Adam	Adam
Learning rate	0.001	0.001	0.001	0.001
Activation function of hidden layers	tanh	ReLU	tanh	ReLU

**Table 4:** Comparison of performance of the soft sensor models on unseen test data.

Model		Model type	RMSE	NRMSE	RMSPE	KGE	Correlation coefficient	Standard deviation
A	CGB with an LSTM neural network as the black-box component	Grey box	5.1743	0.0023	0.46%	0.9992	0.9999	364.8
B	CGB with an MLP neural network as the black-box component	Grey box	5.0394	0.0022	0.45%	0.9994	0.9999	364.8
C	LSTM neural network	Data driven	6.0134	0.0027	0.52%	0.9994	0.9999	365.2
D	MLP neural network	Data driven	5.5287	0.0025	0.49%	0.9988	0.9999	364.6
E	RBF neural network optimised with DE (Deng et al.^37^)	Data driven	–	–	9.35%	–	–	–

According to the RMSE, NRMSE, and RMSPE error metrics presented in Table 4, the predictive performance of the models increases in the order: B, A, D, and C. Both grey-box models exhibit better performance than the data-driven models. The CGB Model B demonstrated superior predictive performance compared to the data-driven Models D and C, achieving reductions in RMSE values by 8.9% and 16.2%, respectively. Additionally, Model B matches the highest KGE value (0.9994) among the models developed in this study, indicating robust predictive capabilities. The KGE metric represents a combination of correlation, bias, and variability of the model. KGE values close to 1 indicate excellent model performance, and hence, the KGE metric also confirms the excellent performance of model B. Moreover, the standard deviation of 364.8 obtained from Model B predictions is the closest among all models to the standard deviation of 364.9 observed in the measured melt viscosity values. This suggests that Model B most effectively captures the variability inherent in the observed data, outperforming the other models. All these performance metrics confirm that the CGB Model B has the best predictive performance.

The superior performance of the grey-box models relative to the fully data-driven models can likely be attributed to the incorporation of the physics-based framework within the CGB models. As shown in Fig. 9a, the physics-based component employed in the CGB model enables the soft sensor to capture the dynamics accurately (despite the large residuals). This likely allows the black-box component of the CGB model to focus on fine-tuning and capturing nonlinearities, which is a simpler learning task than learning the entire input-output relationship from scratch.

The soft sensor models developed in this study can be compared with the previous work by Deng et al.^37^ using the RMSPE metric as both studies utilised the same experimental dataset. It is clear that all models developed in this study show a significant improvement in performance compared to the RBF neural network-based soft sensor by Deng et al.^37^ (i.e., model E). This superior performance of the CGB and data-driven models can be attributed to the deep neural network architectures that can capture complex nonlinear patterns in the data, unlike the RBF neural network used in the previous work^37^.

**Fig. 10:**
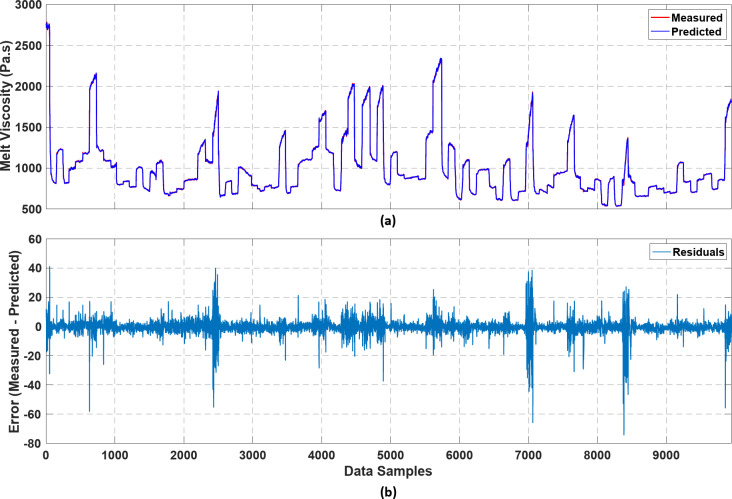
Performance of the CGB model with an LSTM neural network as the black-box component (i.e., Model A): (**a**) comparison of CGB model predictions with experimentally measured melt viscosity values in the test set (**b**) residual plot for the CGB model.

**Fig. 11:**
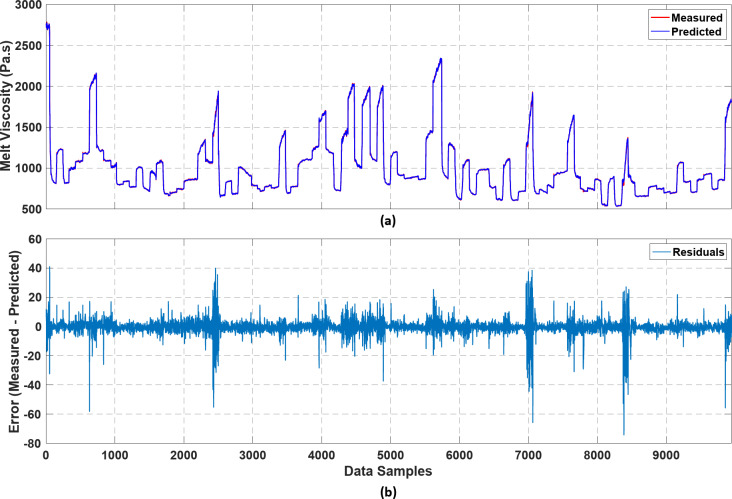
Performance of the CGB model with an MLP neural network as the black-box component (i.e., Model B): (**a**) comparison of CGB model predictions with experimentally measured melt viscosity values in the test set (**b**) residual plot for the CGB model.

As the CGB models were found to have superior performance compared to fully data-driven models, they were further analysed by plotting the CGB model predictions against the experimentally measured melt viscosity values along with model residuals for each data sample in the test set. These plots corresponding to Models A and B are visually presented in Figs. 10 and 11, respectively.

It is clear from both Figs. 10 and 11 that both grey-box soft sensor Models A and B can accurately track the experimentally measured melt viscosity values across the entire processing range of the extruder. When comparing Figs. 10 and 11 with Fig. 9, it is obvious that the black-box component of the CGB model was able to bring down the significant prediction errors in the SGB component resulting in excellent prediction accuracy. The residual plots presented in Figs. 10b and 11b indicate that a majority of the prediction errors made by both the grey-box and data-driven soft sensor models are well within a magnitude of 50. There are a few prediction errors with magnitudes greater than 50, and these large prediction errors are mostly present where screw speed step changes were made during data collection. Among the 9945 data samples in the test set, Model B demonstrated prediction errors exceeding a magnitude of 50 in only three instances, with the largest error magnitude being 65.36. In comparison, Model A exhibited prediction errors exceeding a magnitude of 50 in seven instances, with the largest error magnitude reaching 74.29.

These results confirm that the CGB model with an MLP neural network as the black-box component shows excellent predictive performance. The enhanced predictive performance and interpretability of the proposed CGB model should enable real-time monitoring of melt viscosity, which in turn will enable process optimisation and control. Optimisation of extrusion processes will make it possible to improve product quality while reducing power consumption.

## Conclusions

In this study, a soft sensor with a CGB model architecture was proposed to inferentially estimate the melt viscosity of polymer melts in single-screw polymer extrusion processes. The proposed soft sensor incorporates an SGB component combined with a parallel black-box component. The SGB component comprises a physics-based mathematical model fine-tuned with linear regression and predicts the melt viscosity using shear rate and melt temperature as inputs. A deep neural network was used as the parallel black-box component, which compensates for the prediction errors of the SGB component, using extruder process parameters (i.e., barrel set temperatures and screw speed) as inputs.

As the black-box component of the grey-box model, two deep neural network architectures were compared: an MLP neural network and an LSTM neural network. The CGB model with an MLP neural network exhibited the best predictive performance. It was also compared against two fully data-driven models with MLP and LSTM neural network architectures. The CGB model with an MLP neural network recorded the lowest RMSE, NRMSE, and RMSPE metrics of 5.0394, 0.0022, and 0.45%, respectively, outperforming both data-driven models. Furthermore, this CGB model exhibited reductions of 8.9% and 16.2% in terms of the RMSE values compared to the data-driven models based on MLP and LSTM neural networks, respectively. This confirms that the integration of the physics-based model has enabled the soft sensor to capture the dynamics in the process accurately, simplifying the learning task of the parallel-black box component. The performance of the CGB model was further compared against a soft sensor based on an RBF neural network reported in a previous study. The CGB model showed an increase of approximately 95% in terms of predictive performance compared to the soft sensor reported in the previous work.

The high accuracy reported by the grey-box soft sensor model and its ability to inferentially estimate the melt viscosity in real time without disrupting the melt flow make it an attractive solution for the polymer processing industry. Furthermore, the proposed soft sensor model can be used to optimise and control polymer extrusion processes. The main limitations of this work are that the soft sensor cannot detect viscosity changes due to changes in material properties and is not adaptive to changes in the polymeric material being processed. Future research should focus on addressing these limitations.

## Data Availability

The datasets used during the current study are available from the corresponding author on reasonable request.
